# Placebo support: A possible weaning trial in ventilator dependency

**DOI:** 10.4103/0972-5229.68213

**Published:** 2010

**Authors:** Abhishek Bansal, Anurag Tewari, Harsimran Singh, Shuchita Garg, Abhinav Sharma

**Affiliations:** Department of Anesthesiology and Resuscitation, Dayanand Medical College and Hospital, Ludhiana, Punjab, India; 1 Department of Critical Care Medicine, Dayanand Medical College and Hospital, Ludhiana, Punjab, India

Dear Editor,

Successful weaning is defined as effective spontaneous breathing without any mechanical assistance for 24 hours or more.[1]

A 22-year-old male presented with quadriplegia, dyspnea, and hypotension after Road traffic accident (RTA). Trachea was intubated using Manual in line stabilization (MILT). MRI C spine indicated gross edema and contusions at C4-C7 level. He was put on SIMV mode. Routine blood analysis, CT head and ultrasound abdomen were normal.

Over the next three days his general condition improved. The patient had a MAP above 90mmHg and TLC 8000/cm^3^. His hemodynamic and renal parameters were normal. Repeat MRI (4^th^ day) showed marked improvement as the cord edema decreased. The extent of quadriplegia however remained.

Weaning was initiated using the standard weaning protocol. Initiation of weaning was started by putting the patient on pressure support ventilation of 15 cm H_2_O and PEEP of 4 cm H_2_O. The pressure support was gradually decreased to 3 cm of H_2_O and PEEP was decreased to 0 cm.

He remained comfortable on spontaneous mode and vitals were satisfactory. The ABG showed PO_2_ = 108, SO_2_ = 98, PH = 7.365, PCO_2_ = 33. The ventilatory graphics showed spontaneous tidal volume = 400-500 ml, spontaneous RR 16-20/min, minute ventilation = 6.4-8l/min., compliance 40-45 ml/cm of H_2_O. His rapid shallow breathing index was found to be (f/vt) = 45 breaths/min/l.

We decided to put the patient on a T-piece. On the T-piece, he would become restless and would frantically gesture demanding renewed ventilatory support. He would start having marked tachycardia and tachypnea with anxious looks pointing us with hand to put on the ventilator. On 7^th^ day, tracheostomy was done anticipating prolonged need for tracheal intubation. NCV studies did not show any diaphragmatic involvement.

As the ventillatory graphics and ABG were pointing towards a successful weaning, we suspected psychological dependency. Psychiatry consultation was sought but proved to be of no help. We decided to do a ‘sleep separation test’. At night he was given midazolam and when the patient was asleep the ventilator was removed and he was put on a T piece. The patient remained comfortable with no alteration in vital signs. Patient was under strict monitoring. ABG done after 30 min was normal. He was comfortable the whole night. When he woke up in the morning, after seeing he was disconnected from the ventilator he again got tachypneic and made gestures to be attached to ventilator. As he became very restless he was put on ventilator.

Suspecting ventilator dependency we decided to put the patient on “placebo ventilation”. Consent was obtained from the attendants.

**Figure 1:**
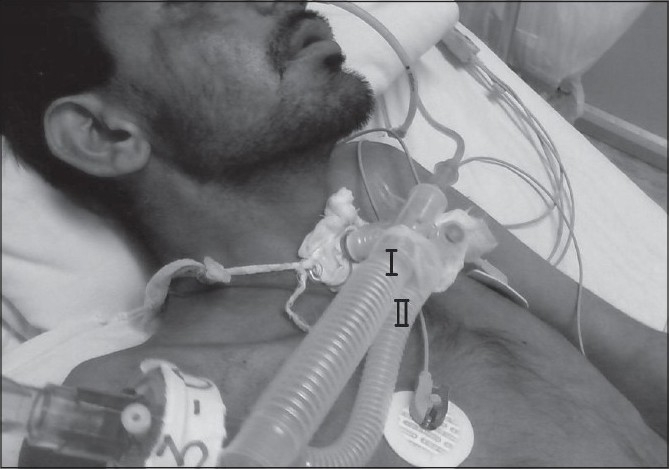
(I) T piece attached to the tracheostome (II) Ventilator circuit tied to the T piece

We camouflaged his breathing system by putting him on T-piece with ventilator circuit tied to it so as to give a fallacious impression of being ventilated by the ventilator [Figure 1], while in fact he would be generating his own breaths.

He was put on similar placebo ventilation multiple times with incremental time duration. He remained calm and continued to breathe on his own. ABGs after 30 min of such ventilation were normal. After two days of observation, we gained his confidence by revealing the truth and he was convinced that he could breathe on his own. Successful separation from the ventilator was achieved.

Jubran A[2] has elaborated upon why patients are ventilator-dependent. These factors require both clinical awareness as well as focused assessment. The search for underlying causes for ventilator dependency is pertinent if previously unrecognized but reversible conditions are discovered. Understanding the determinants of respiratory failure and ventilator dependence is a vital step in providing quality care. We could help prevent prolonged ICU stay and financial burden in our patient by the use of our placebo technique.
